# Detective quantum efficiency of electron area detectors in electron microscopy

**DOI:** 10.1016/j.ultramic.2009.04.002

**Published:** 2009-08

**Authors:** G. McMullan, S. Chen, R. Henderson, A.R. Faruqi

**Affiliations:** MRC Laboratory of Molecular Biology, Hills Road, Cambridge CB2 0QH, UK

**Keywords:** DQE, MTF, CMOS detector, CCD, MAPS, Film, Medipix, Electron backscatter

## Abstract

Recent progress in detector design has created the need for a careful side-by-side comparison of the modulation transfer function (MTF) and resolution-dependent detective quantum efficiency (DQE) of existing electron detectors with those of detectors based on new technology. We present MTF and DQE measurements for four types of detector: Kodak SO-163 film, TVIPS 224 charge coupled device (CCD) detector, the Medipix2 hybrid pixel detector, and an experimental direct electron monolithic active pixel sensor (MAPS) detector. Film and CCD performance was measured at 120 and 300 keV, while results are presented for the Medipix2 at 120 keV and for the MAPS detector at 300 keV. In the case of film, the effects of electron backscattering from both the holder and the plastic support have been investigated. We also show that part of the response of the emulsion in film comes from light generated in the plastic support. Computer simulations of film and the MAPS detector have been carried out and show good agreement with experiment. The agreement enables us to conclude that the DQE of a backthinned direct electron MAPS detector is likely to be equal to, or better than, that of film at 300 keV.

## 1 Introduction

For many decades, film has been the recording medium of choice for electron microscopy. However, the need to have immediate access to the resulting images and the demand to be able to use much lower electron dose has meant that most users would prefer a recording medium with lower, or zero, noise level and the immediate availability of a digital image. While currently available phosphor/fibre-optic charge coupled device (CCD) cameras do provide more immediate readout and much lower noise than film, their performance near the Nyquist frequency when operating above 120 keV, measured in terms of detective quantum efficiency (DQE) and modulation transfer function (MTF), is less than that of film. Detectors based on new technology promise improved DQE and MTF as well as the ability to acquire data in different ways. For example in single particle electron cryomicroscopy (cryoEM), it is believed that image resolution is limited by image blurring caused by charging and movement of the specimen during the low dose exposure [1]. New detectors may allow better final images to be obtained from dose-fractionated series in which multiple lower dose images are added together after the removal of inter-image shifts [2–4].

We have measured under the same conditions the DQE and MTF of four electron area detectors: Kodak SO-163 film, a TVIPS 224 CCD detector, the Medipix2 hybrid pixel detector [5,6], and a direct electron monolithic active pixel sensor (MAPS) detector [7,8]. The first two are well established commercial products while the latter two are experimental detectors on which future commercial detectors might be based.

## 2 Background

The MTF measures the response of a detector to a signal of fixed amplitude as a function of spatial frequency. By definition the value of the MTF at zero spatial frequency is unity but in the presence of long range scattering within the detector this can fall rapidly with increasing spatial frequency. As the detectors studied here have different pixel sizes, we measure the spatial frequency, ω, in units of the Nyquist frequency given by the reciprocal of twice the pixel spacing. In the case of film the pixel spacing is defined by the densitometer. The response of a perfect pixel detector is governed by the integral over a pixel which results in a damping of the response with increasing spatial frequency given by sinc(πω/2)≡sin(πω/2)/(πω/2). While it is desirable to have a high MTF, in applications such as cryoEM or cryo-tomography, where the total dose is strictly limited by radiation damage, it is vital to have as high a DQE as possible.

The DQE, defined as the ratio of the square of the output signal to noise ratio ${\left(S/N\right)}_{\mathrm{out}}$ to the square of the input signal-to-noise ratio ${\left(S/N\right)}_{\mathrm{in}}$:

(1) $$\mathrm{DQE}={\left(S/N\right)}_{\mathrm{out}}^{2}/{\left(S/N\right)}_{\mathrm{in}}^{2}$$

provides a measure of the quality with which incident electrons are recorded. A perfect detector has a DQE of unity and to achieve this all incident electrons must be detected with equal weight. The DQE of real detectors is always smaller than unity reflecting the fact that in practice incident electrons are recorded with different weights. The noise terms in Eq. (1) relate to the stochastic noise associated with a pixel. In general this is larger than the associated variance in an image and only equal to the variance when there is no inter-pixel mixing, i.e., when signals in different pixels are independent. The generalisation of Eq. (1) to non-zero spatial frequency, DQE(ω), associates the noise terms with the corresponding power, or Wiener spectrum, W(k). For a one dimensional signal $\left\{x_{j}\right\}$ over pixels j=0,1,…,N-1 we use the definition

(2) $$W(k)=\frac{1}{N}{\left|\overset{N-1}{\underset{j=0}{\sum }}x_{j}e^{-2\pi \mathrm{ijk}/N}\right|}^{2}$$

so that an uncorrelated random sequence of unit amplitude Poissonian events with a mean of n events per pixel has W(k)=n for all k. Calculation of the DQE for an area detector uses the noise power spectrum, NPS(ω), given by the circular average of the two dimensional generalisation of Eq. (2). For an incident beam obeying Poisson statistics, with on average n electrons per pixel and giving an average output signal of $d_{n}$ (so that that the average signal per primary electron is $d_{n}/n$)

(3) $$\mathrm{DQE}(\omega )=\frac{d_{n}^{2}{\mathrm{MTF}}^{2}(\omega )}{n{\mathrm{NPS}}_{n}(\omega )}$$

(see [9–11] and references therein). As MTF(ω)→1 as ω→0,

(4) $$\mathrm{DQE}(0)=d_{n}^{2}/n{\mathrm{NPS}}_{n}(0)$$

Introducing a normalised noise power spectrum, N(ω), defined by

(5) $$N(\omega )={\mathrm{NPS}}_{n}(\omega )/{\mathrm{NPS}}_{n}(0)$$

and using Eq. (4) enables Eq. (3) to be rewritten as

(6) $$\mathrm{DQE}(\omega )=\mathrm{DQE}(0)\frac{{\mathrm{MTF}}^{2}(\omega )}{N(\omega )}$$

which explicitly shows the origin of the spatial frequency dependence in DQE(ω).

While the output signal at a given frequency ω is proportional to MTF(ω), in a pixellated detector the noise includes the contributions at ω and all aliased frequencies, i.e., $\omega_{i}=\omega +2i$ for i=±1,±2,…. The aliased contributions in N(ω) result in a decrease in DQE(ω) with increasing spatial frequency. In a perfect pixel detector DQE(0)=1, MTF(ω)=sinc(πω/2) and since

(7) $$\overset{i=\infty }{\underset{i=-\infty }{\sum }}{\mathrm{sinc}}^{2}\left[\frac{\pi }{2}(\omega +2i)\right]\equiv 1$$

N(ω)=1. Putting these into Eq. (6) gives

(8) $$\mathrm{DQE}(\omega )={\mathrm{sinc}}^{2}(\pi \omega /2)$$

and DQE(Nyquist) for a perfect pixel detector is ${\left(2/\pi \right)}^{2}=0.405$.

**Fig. 1:**
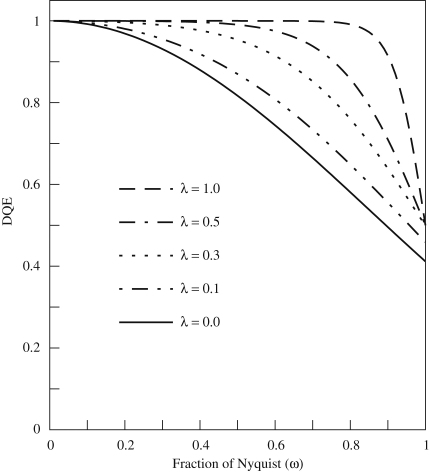
Illustration of the effect on the spatial frequency dependence of the DQE resulting from damping the MTF via deterministic blur. The blur is given by a Gaussian with 1/e length parameter, λ, specified in terms of the pixel pitch (see Section 3). λ=0 corresponds to a perfect pixel detector with no blur and $\mathrm{DQE}(\omega )={\mathrm{sinc}}^{2}(\pi \omega /2)$. The MTF at the Nyquist frequency varies as $\left(2/\pi )\;\exp(-\pi^{2}\lambda^{2}/4\right)$ and the corresponding values with λ equal to 0, 0.1, 0.3, 0.5 and 1.0 are 63.7%, 62.1%, 51.0%, 34.4% and 5.4%, respectively.

The reduction in Nyquist DQE due to noise aliasing means that it can actually be advantageous for a detector to have a lower MTF at high spatial frequency, provided that the reduction in MTF results from deterministic blur, i.e. it can be described by a unity-gain linear filter. In the absence of aliasing, deterministic blur does not affect the DQE [12] as the signal and noise terms are damped equally. The damping will however be greater for the aliased noise terms at higher frequency and so the relative reduction in their contribution leads to an increased DQE at higher spatial frequencies. This is illustrated by the calculation shown in Fig. 1 where the expected DQE is plotted as a function of spatial frequency for various amounts of deterministic blur in an otherwise perfect pixel detector. As the blurring reduces the signal, having low readout noise is essential. Although it can produce a useful improvement in DQE at 0.75 Nyquist, the DQE at Nyquist is always limited to at most 50%. It is important to stress that this result only applies if the reduction in MTF is due to deterministic blur. The effect will also be less when the input signal has an intrinsic width and hence a natural damping of higher spatial frequency components such as when the input signal is better described by a Gaussian with non-zero width rather than a delta function.

The fall in DQE of a detector with spatial frequency forces a trade-off between field of view and spatial resolution. For example, at 300 keV the DQEs of current fibre-optic coupled CCDs are good at low spatial frequency but fall rapidly with increasing spatial frequency. To study higher spatial resolution features and yet maintain a high output signal-to-noise ratio, it is therefore necessary to use higher magnification, possibly accompanied by adjacent pixel binning. This results in a decreased field of view and number of effectively independent pixels. In the study of radiation sensitive samples, the dose that can be used is limited and so any fall-off in DQE(ω) fundamentally restricts the utility of a detector.

The use of electron microscopes with higher energies has a number of advantages including: greater penetration; reduced influence of charging; simpler interpretation of images due to less multiple scattering; flatter Ewald sphere; and reduction in distortions from lens aberrations at shorter wavelength. In current commercial microscopes, practical considerations limit the upper energy to 300 keV. The energy of a 300 keV electron is $∼10^{7}k_{B}\;T$ and so there would seem to be no fundamental reason why an electron area detector should not approach perfection. However as the operating voltage of an electron microscope increases above 100 keV, the efficient detection of electrons becomes more problematic and the choice of electron detector more important.

Electrons interact with matter very strongly but lose energy relatively slowly and at a rate that increases as the energy of the incident electron decreases. As a result the trajectories followed by incident electrons are highly stochastic and have an effective range that increases rapidly with incident energy. For example the path of a 300 keV electron in silicon can easily exceed 400μm. Moreover the signature, in terms of deposited energy, left by the electron has a built-in bias away from the initial point of incidence. Consequently it is difficult to build a detector with small pixels within which it is possible to capture a sizeable fraction of the incident energy. Using larger pixels helps but the actual pixel size required with incident 300 keV electrons would limit the total number of pixels in a detector. The range of the incident electrons can be reduced by using higher Z materials such as CdTe. Unfortunately with higher Z materials the fraction of incident electrons that effectively bounce off the detector before leaving a significant signal, would also be increased.

The rate of energy loss by incident electrons is sufficient to leave detectable signals in a sensitive layer having a thickness of only a few μm, e.g., the emulsion layer of film or the epilayer in a MAPS detector. It is possible for a normally incident high energy electron to scatter into the plane of a sensitive layer that is only a few μm thick but in general they will pass straight through leaving a spatially well defined signal. Having passed through the sensitive layer an electron will travel into any supporting matrix from which it can scatter back through the sensitive layer again. As the rate of energy loss increases with decreasing energy, the additional signal can be stronger than the initial signal. At zero spatial frequency the increase in average signal helps mitigate any reduction in DQE that might be expected from the additional variance in total signal associated with backscatter. With increasing spatial frequency the contribution from backscattered electrons to the signal decreases much faster than the corresponding contribution to the noise. Electron backscattering will therefore result in a drop in DQE with increasing spatial frequency.

Even if electron backscatter is removed entirely, the actual amount of energy deposited in a thin detector is still governed by the stochastic nature of the interactions. This intrinsic variance in the deposited energy sets an upper limit to the DQE for any detector that responds linearly to the deposited energy. Most detectors are not truly linear in energy but have an upper limit to their response, while detectors, such as film, principally respond to the number of incident electrons and not the energy deposited. In film, a grain is either sensitised or not and the binary nature of the grain sensitisation helps to reduce the variance in response to incident electrons. However, there is still a variation in the number of grains that are sensitised by an individual electron.

Film is still the detector of choice for applications such as high resolution cryoEM, where problems such as lack of dynamic range, inconvenience in handling and the need for post-processing are more than outweighed by the increased quality of the data that can be obtained thanks to its unique combination of high MTF, DQE, and field of view. To make the most of these, film must however be used with an optimal exposure of $∼1e/\mu m^{2}$. The DQE(0) for film at 100–120 keV is known to be approximately 75% and drops with increasing energy so that by 300 keV it is down to 50% [13–17]. Unlike electronic detectors, film does not have a natural pixel size. The diameter of a developed grain is of the order of 1μm but these are not placed on a uniform grid. The pixel size, and hence Nyquist frequency, with film is imposed by the densitometer. In cryoEM work this is typically ∼10μm. We were unable to find published results for the DQE(ω) of film. The presence of backscatter from film holders is also well known but poorly characterised. We therefore modified a number of standard metal-backed Tecnai film holders to include a rectangular cut-out window.

A number of previous publications have presented measurements of the performance of CCD detectors. There are useful reviews by De Ruijter [10] and Faruqi and Subramaniam [18]. Zuo [17] made a careful measurements of DQE(0) on an early Gatan CCD detector, giving values of 0.7 (120 keV) and 0.46 (400 keV) for CCD compared with ≈0.73 for SO-163 film at 100 keV. Meyer and Kirkland [19] used the formulation of De Ruijter [10] to measure DQE(0) and DQE(Nyquist) for an early Gatan CCD detector with a 24μm pixel size at 35% and 2%, respectively, for 400 keV electrons and 87% and 20%, respectively, for 100 keV electrons.

In the study of biological molecules using electron cryomicroscopy, Zhang et al. [20] showed that at 120 keV, the performance of film using a 7μm pixel and the Gatan Ultrascan 4000SP with a 15μm pixel were very close. Booth et al. [21] showed, first at 200 keV, then at 300 keV [22], that the signal-to-noise ratio of the Gatan US4000 CCD camera exceeded film below 40% of Nyquist at 200 keV and below 20% of Nyquist at 300 keV, with film being better at higher resolution, even though the exposure level they used on film produced an optical density (OD) significantly less than 1.0. Finally, Sander et al. [23] showed at 200 keV that the signal-to-noise ratio from processed single particle images was better on a TVIPS F415 CCD with 15μm pixels up to 20% of Nyquist, with film using an 8μm digitisation step again being superior at higher spatial frequency. They recommended adjacent 2×2 or 4×4 pixel binning to ensure the acquisition of superior CCD images but, of course, this greatly reduces the field of view. Overall, the current consensus is that phosphor/fibre optics/CCD detectors are very valuable but that for optimal work at high resolution, film is still advantageous.

The Medipix2 is a hybrid pixel detector [5,6,24] in which each $55\times 55\;\mu m^{2}$ pixel of a 300μm thick silicon sensor layer is bump-bonded to detector electronics on a separate ASIC chip. The electronics associated with each pixel consists of 504 transistors and amplifies the pulses from individual incident electrons, filters them through an energy discriminator and counts selected events (up to $2^{13}$ per pixel) within the selected energy window. After a set time the whole image is read out, the counters reset to zero and counting recommenced. The Medipix2 design ensures zero noise, good resolution and no radiation damage to the detector at electron energies up to 250 keV. At 120 keV the Medipix2 is as good as, or better than, film, albeit with a much larger pixel size. At higher energies the performance rapidly degrades as the range of the primary electrons increases. Moreover, above 250 keV, the primary electrons can penetrate the detector layer and damage the underlying electronics.

The other type of detector we have tested is a direct electron MAPS detector [7,8,25,26]. In this case, electron-hole pairs are generated along the path of a primary electron in a thin, lightly doped silicon epilayer. The resulting signal is expressed as a voltage drop across a capacitor formed by a reverse biased diode fabricated to extend into the epilayer. The sensor is read out continuously via a rolling shutter mode in which rows of the sensor are successively selected and the capacitor voltages along the selected row read out and reset. In this mode the detector has very little dead time and, in principle, can be read out at any rate depending on the number of analogue to digital converters associated with the columns that are either embedded in the chip or located nearby. Since the primary electrons are directly incident on the MAPS detector, the diodes and three or four transistors present on each pixel must be radiation hardened. Existing MAPS detectors such as the FillFactory STAR250 [27] show that this is possible but the MAPS detector tested here was not rad-hard.

We do not present results for image plates [17,28]. Their sensitivity and large linear dynamic range make them ideal detectors for some applications in electron microscopy. At higher incident energies the density and thickness of the phosphor layer will result in a relatively high zero spatial frequency DQE. However the stochastic scattering of electrons in the phosphor layer will degrade performance at finite spatial frequencies. Image plates also have a large effective pixel size and require a separate time consuming digitisation step.

## 3 Methods

All the images were taken on a Tecnai F30 microscope fitted with a Faraday cup that used a commercial picoammeter (Agar, Probe Current Meter) to measure the beam current. The MTF was measured from electron shadow images of a straight edge, consisting of a 0.5 mm gold or 0.2 mm platinum wire mounted at the pointer position. The MTF can be measured by other methods [29–31] but the straight edge method [9,30] used here enables the MTF to be measured accurately down to low spatial frequencies. The DQE was obtained using the MTF results and flat field images taken with known average dose per pixel. For Kodak SO-163 film, the dose was adjusted to give an optical density of approximately 1 after development for 12 min in full strength D19 developer at $20{\;}^{\circ }C$. For the other detectors the dose was not so important. As the Medipix detector has no noise it was possible to decrease the dose so that only a few hundred electrons were incident on each frame. In this way, the statistics of the individual events could be counted and the DQE independently estimated [6].

In calculating MTF the image of the edge is first examined to ensure it is straight and featureless. Suitable areas are selected that extend far enough away from the edge to allow bright and dark field values, b and d, respectively, to be determined. Areas containing unwanted features are masked out and not used in subsequent analysis. The edge spread function, ESF(x), is calculated as

(9) ESF(x)=(〈n(x)〉-d)/(b-d)

in which x is the perpendicular distance of a pixel from the fitted edge and 〈n(x)〉 is the average value of the signal in the pixels at distance x. Differentiating ESF(x) with respect to x gives the line spread function, LSF(x) whose Fourier transform is the modulation transfer function. Rather than differentiating the noisy ESF(x), we prefer to fit a model from which the MTF can be deduced.

Various functional forms can be used where the relationships between the LSF(x), ESF(x), MTF and underlying point spread function, PSF(r), are known either analytically or numerically [9]. We have found a normalised, linear combination of a small number of Gaussian functions convenient. For a Gaussian, specified by a length parameter λ, the relationship between the various functions is known analytically and given by

(10) $${\mathrm{PSF}}_{G}(r)=\exp(-r^{2}/\lambda^{2})/\pi \lambda^{2}$$

(11) $${\mathrm{LSF}}_{G}(x)=\exp(-x^{2}/\lambda^{2})/\pi \lambda$$

(12) $${\mathrm{ESF}}_{G}(x)=\mathrm{erfc}(-x/\lambda )/2$$

(13) $${\mathrm{MTF}}_{G}(\omega )=\exp(-\pi^{2}\lambda^{2}\omega^{2}/4)$$

where distances are measured in units of the pixel pitch and the spatial frequency, ω, is expressed in terms of fraction of Nyquist. The effects of integration within a pixel can be combined with a Gaussian point spread model to give

(14) $$\mathrm{MTF}(\omega )=\mathrm{sinc}\left(\frac{\pi }{2}\omega \right)\exp(-\pi^{2}\lambda^{2}\omega^{2}/4)$$

and

(15) $$\mathrm{ESF}(x)=\frac{\lambda }{2}\underset{\sigma =\pm 1}{\sum }\sigma \left(t_{\sigma }\mathrm{erfc}(-t_{\sigma })-\frac{1}{\sqrt{\pi }}\exp(-t_{\sigma }^{2})\right)$$

in which $t_{\sigma }=(x+\sigma /2)/\lambda$ and σ=±1.

**Fig. 2:**
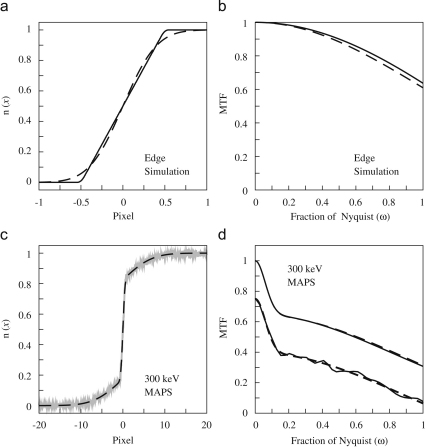
Illustration of ESF and MTF calculations. (a) shows the calculated ESF (solid) from a simulated image of a perfect pixel detector and the corresponding fits based on models using a single Gaussian (dashed). The fit obtained using a single Gaussian with sinc correction is not shown as it is indistinguishable from the calculated ESF result. (b) shows the MTF results of a perfect detector sinc((π/2)ω) MTF (solid) and that obtained from the single Gaussian fit (dashed). The MTF of the single Gaussian with sinc correction is indistinguishable from the perfect detector result. (c) shows the calculated ESF (grey) calculated from the measured 300 keV edge image of the MAPS detector and corresponding fit based on a double Gaussian model (dashed). (d) compares the calculated MTF obtained from the double Gaussian fit (dashed) and double Gaussian fit with sinc correction (solid). Also shown (but offset by -0.25 vertically) is a comparison of the MTF from the double Gaussian fit (dashed) and that calculated via the numerical differentiation of the ESF (solid).

Calculation of the MTF based on a fit to ESF is illustrated in Fig. 2, which shows the results from a simulated edge image of a perfect pixel detector and the measured edge image at 300 keV recorded on the MAPS detector. The fit to simulated ESF as shown in Fig. 2a, is not improved by using more than a single Gaussian. The fit obtained using a single Gaussian with Eq. (15), is indistinguishable from the ESF and not shown. The calculated MTF is similarly indistinguishable from that of a perfect pixel detector.

For real detectors the inclusion of the explicit pixel integration factor as in Eq. (15), typically makes very little difference to the final MTF. This is illustrated in Fig. 2c, which shows the ESF and corresponding fits generated from edge images at 300 keV obtained using the MAPS detector. A double Gaussian model was used and the resulting fits specified in terms of (*amplitude*, *length scale*) using Eq. (12) are (0.350, 7.68) and (0.650, 0.551) while those using Eq. (15) are (0.354, 7.59) and (0.645, 0.318). Both fits give a very good description of the ESF, though the parameters obtained using Eq. (15) are presumably more directly related to intrinsic physical properties of the detector. As expected from the similarity of the ESF fits, the corresponding MTF curves as shown in Fig. 2d are also similar.

The number of terms needed to fit the ESF is found empirically by monitoring the difference between the ESF and the optimised fit. In all the cases studied here, no more than 4 terms were needed, which justifies the method and choice of fit.

As a further check the MTF obtained via numerical differentiation of the ESF(x) is also calculated. This is free of model bias but much noisier. It is however possible to estimate the noise level since the intermediate LSF(x), resulting from differentiation of the ESF(x), is an even function. As a result the MTF signal in the Fourier transform of the LSF(x) has a well defined phase and an estimate of the noise is provided by the quadrature signal.

DQE(ω) is calculated via Eq. (3) using the measured MTF and flat field images in which the number of electrons per pixel is known. The NPS(ω) is calculated from the FFT of the flat field images. As it is difficult to obtain an accurate measurement of the low frequency NPS(ω), the value of DQE(0) is estimated independently using Eq. (1). As noted earlier in Section 2, the signal from an incident electron is not in general constrained to a single pixel and so the variance in an image will underestimate the true noise per pixel. To correct for this we look at the estimated noise, $S_{n}^{2}$, in n×n binned images with increasing n. As n increases the relative contribution from neighbouring pixels to the variation seen in a binned pixel decreases and in the limit of large n, $S_{n}^{2}$ reduces to the sum of $n^{2}$ (assumed independent) contributions from the original pixels. The noise per pixel is found by plotting $S_{n}^{2}/n^{2}$ as a function of n which should initially increase with n but then plateau at the required value.

**Fig. 3:**
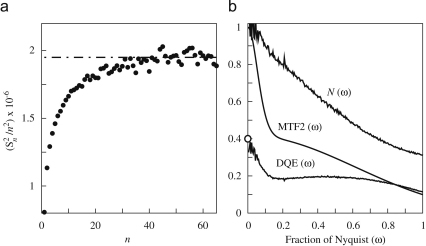
(a) Illustration of the noise binning method for calculating DQE(0) by plotting the estimated noise, $S_{n}^{2}/n^{2}$, as a function of n-fold binning. Results (black circles) are from the difference between successive images captured on the MAPS detector at 300 keV. The plateau value of $1.97\times 10^{6}$ illustrated by the horizontal dot-dashed line represents twice the noise per pixel. The average signal was 1770 and there were 7.9 electrons per pixel and so $\mathrm{DQE}(0)=1770^{2}/(\frac{1}{2}1.97\times 10^{6})/7.9=0.40$. (b) shows the calculated DQE(ω) of the MAPS detector at 300 keV along with the N(ω) and ${\mathrm{MTF}}^{2}(\omega )$ illustrating the origin of the spatial frequency dependence of DQE. DQE(ω) was calculated using Eq. (6) and the value at ω=0 calculated independently in (a) is indicated (∘).

It is convenient to work with the difference between successive frames as doing so naturally suppresses effects from imperfections in both the dark field correction and the illumination that would otherwise lead to a linear ramp rather than a plateau. Assuming the noise in the successive frames is independent, the resulting plateau value will however be twice the required value. The process is illustrated in Fig. 3 using images obtained with the MAPS detector at 300 keV. While using the difference between successive frames works well for electronic detectors, it will give an over-optimistic estimate of the DQE compared with what can be achieved using the normal flat field corrected images and cannot be used with single shot detectors such as film. In order to obtain reliable results with film it is important to use fresh developer and avoid areas with drying marks, scratches and dust in the analysis.

The calculated DQE(ω) of the MAPS detector at 300 keV along with ${\mathrm{MTF}}^{2}(\omega )$ and N(ω) contributions as used in Eq. (6), are shown in Fig. 3b. The value for DQE(0) calculated using the extrapolated noise per pixel from Fig. 3a is also shown and the value, 0.40, can be seen to be in good agreement with the zero frequency limit of the DQE(ω) curve. The sharp drop in the DQE at low spatial frequency is associated with a rapid drop in the ${\mathrm{MTF}}^{2}(\omega )$ that is not reflected in the NPS(ω). The simulations presented in Section 6.2, support the interpretation of this drop in DQE being associated with backscattering of electrons from the substrate of the detector.

The computer programs used in the analysis are available from the author (G.M.).

## 4 Comparison of film, TVIPS CCD, Medipix2 and MAPS detectors

**Fig. 4:**
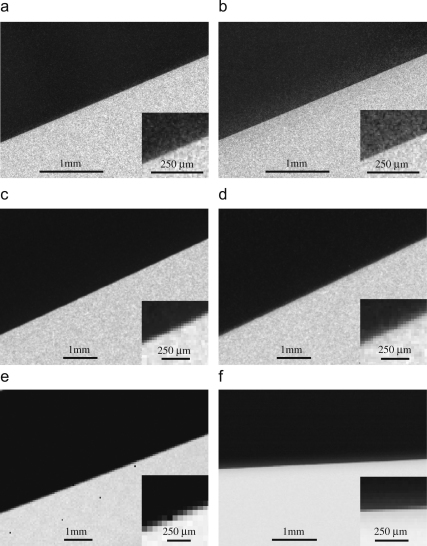
Shadow image of the edge of 0.5 mm gold wire. (a) SO-163 film after exposure to 120 keV electrons, 6μm pixel, 19 electrons/pixel, (b) SO-163 film after exposure to 300 keV electrons, 6μm pixel, 24 electrons/pixel, (c) TVIPS 224 CCD detector, 120 keV electrons, 24μm pixel, 104 electrons/pixel, (d) TVIPS 224 CCD detector, 300 keV electrons, 24μm pixel, 134 electrons/pixel, (e) Medipix2/Quad, 120 keV electrons, 55μm pixel, 3800 electrons/pixel; four dead pixels are visible, and (f) MAPS, 300 keV electrons, 25μm pixel, 24000 electrons/pixel.

**Fig. 5:**
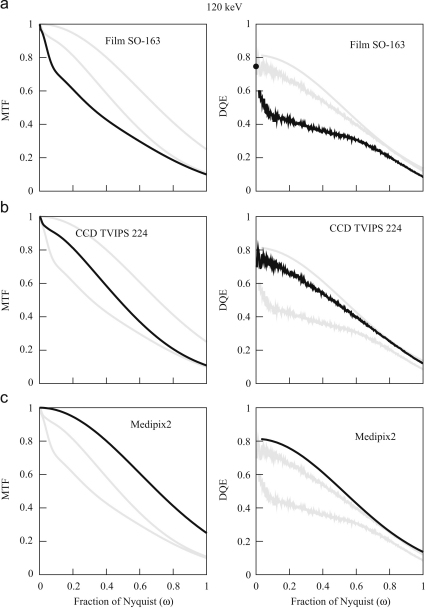
MTF (left) and DQE(ω) (right) calculated from the 120 keV images in Fig. 4 for (a) Kodak SO-163 film, (b) TVIPS 224 and (c) Medipix2 detectors. The indicated detectors results are shown in black but to aid comparison the corresponding results for the other detectors are shown in grey.

**Fig. 6:**
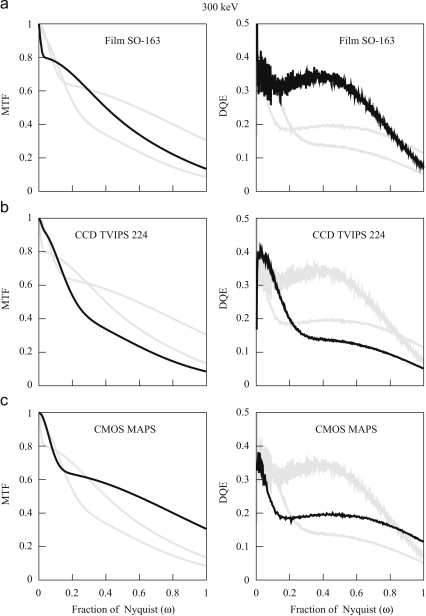
MTF (left) and DQE(ω) (right) calculated from the 300 keV images in Fig. 4 for (a) Kodak SO-163 film using a cut-away holder, (b) TVIPS 224 and (c) MAPS detectors. The indicated detectors results are shown in black but to aid comparison the corresponding results for the other detectors are shown in grey.

Typical images obtained by digitising the optical density on film are shown in Fig. 4a and b. Similar images after bright and dark field corrections of the three electronic detectors are shown in Fig. 4c and d (TVIPS 224 CCD), Fig. 4e (Medipix2) and Fig. 4f (MAPS). Figs. 5 and 6 show the corresponding MTF and DQE(ω) measurements at 120 and 300 keV, respectively. It can be seen that all three detectors are very good at 120 keV. The data shown used pixel sizes of 6μm on film, 24μm on TVIPS and 55μm on Medipix2, but if a larger pixel size is used on film (e.g., 12 or 14μm) and a smaller pixel size on a different phosphor/fibre-optic CCD (e.g., 15μm), then the performance of film exceeds that of the electronic detectors above half Nyquist. The DQE results for the TVIPS CCD and MAPS detector shown in Figs. 5 and 6 were derived using the NPS obtained from the difference between successive frames. If instead, the NPS were obtained from featureless areas of bright and dark field corrected images, even if this flat field correction is carried out immediately before recording an image, the resulting noise levels would be higher and the corresponding DQEs lower. As a result, even at 120 keV with 6μm pixels, film is normally the detector of choice for imaging radiation sensitive specimens.

**Fig. 7:**
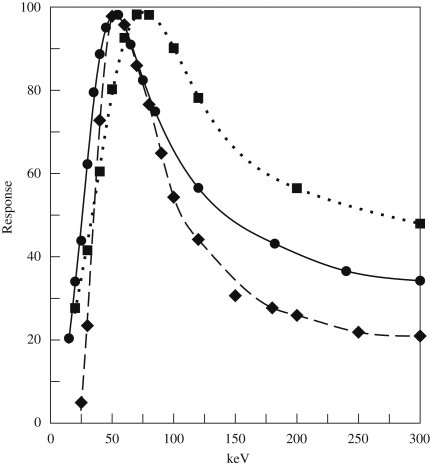
Measured average signal per electron as a function of incident electron energy for TVIPS 224 CCD (■), SO-163 film (•), MAPS (♦) detector. The results have been arbitrarily scaled to have a peak value of ∼100. The lines through the data points are simply aids to the eye. The shape of the response curve is similar for all three detectors. Extrapolating to zero response gives threshold energies of 5, 8 and 24 keV for the CCD, film and MAPS detectors, respectively.

Fig. 7 compares the measured average signal versus incident energy for SO-163 film, the TVIPS 224 CCD detector and the MAPS detector. The curves have been arbitrarily scaled to have peak values of approximately 100. The shapes of the three curves are similar. At very low energies there is a region with no response in which the incident electrons have insufficient energy to reach a detector's sensitive layer. This is followed by a region in which the response increases linearly with increasing incident energy as electrons reaching the sensitive layer deposit all their remaining energy there. At higher energies electrons are able to pass through the sensitive layer and the total energy deposited along the electrons trajectory through the sensitive layer decreases due to the drop in rate of energy loss with increasing energy. The actual shape of the signal versus incident energy curves reflect physical parameters of the detectors. For example the threshold energy required to detect an electron depends on the thickness of any protective passivation layer in a detector, while the position and width of the response peak reflects the thickness of the sensitive layer.

The zero spatial frequency DQE value at 120 keV for film shown in Fig. 5 is similar to that of the TVIPS 224 CCD and Medipix2 detectors, but with increasing spatial frequency the DQE of film drops rapidly. A similar result is illustrated in Fig. 3b where the drop in DQE(ω) can be seen to be associated with a drop in MTF(ω) that is not reflected in a corresponding drop in the noise power spectrum. This interpretation is consistent with Fig. 5a where the observed drop in DQE(ω) up to ω=0.1 matches that of the square of the MTF(ω).

The peak response of film as a function of incident electron energy occurs at ∼50keV (see Fig. 7). Incident electrons with higher energy are able to pass through the emulsion and into the plastic support. Some of these will scatter back to the emulsion and enhance the response of the emulsion. Due to the added scattering the additional signal is not localised. The spread of this contribution depends on the electron range in the plastic substrate. An incident 120 keV electron can easily still have 70 keV on entering the plastic support. With this energy its range is over 20μm and so much greater than the 6μm scanning step. The contribution from electron backscattering from the plastic support will therefore produce a long range tail in the point spread function and results in a drop in MTF(ω) at low spatial frequency. The relative contribution, and hence size of the drop, is enhanced relative to the fraction of electrons backscattered due to the reduced energy and corresponding greater rate of energy loss of electrons returning to the emulsion. Since the range in ω over which the drop in MTF occurs is inversely proportional to the range of electrons in the plastic substrate, increasing the incident electron energy decreases the range in ω. This can be seen by comparing the MTF(ω) of film at 120 and 300 keV shown on left hand sides of Figs. 5a and 6a, respectively. At 300 keV the drop in MTF(ω) is restricted to a range in ω where estimates of the noise power spectrum, and hence DQE(ω), are very noisy. This makes it difficult to see the corresponding drop in DQE as a function of ω. The value of DQE(0) determined by the pixel binning method described in Section 3 is ∼0.5. This together with the observed drop in the MTF(ω) would predict that DQE(ω) should drop to ∼0.32, which is in agreement with the measured result shown on the right hand side of Fig. 6a. The rapid drop in DQE with ω limits the usefulness of DQE(0) as a measure of performance of film in imaging. The value of film as a detector in electron microscopy is clearly illustrated by the fact that at 300 keV the DQE(ω) is above 0.3 to beyond 60% of the Nyquist frequency.

**Fig. 8:**
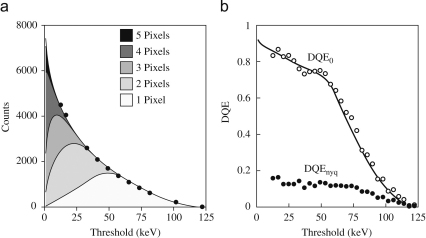
Calculation of the Medipix2 DQE at 120 keV using the statistics of individual electron events. (a) shows the total number of events (•) recorded per frame and the composition in terms of events recorded in a given number of pixels, as a function of the energy threshold. A total of 2100 electrons were expected per frame. For a threshold of one half the incident energy only single pixel events are seen and at 120 keV no events were seen involving six or more pixels even with a very low threshold. (b) shows the DQE calculated using $\mathrm{DQE}(0)={\left(\sum_{i}{\mathrm{iN}}_{i}\right)}^{2}/[(\sum_{i}i^{2}N_{i})(\sum_{i}N_{i})]$, where $N_{i}$ is the number of events in each frame where a single electron is counted in i adjacent pixels. Also shown is the corresponding DQE at Nyquist calculated using Eq. (6).

As the Medipix2 detector counts the events associated with individual electrons it is possible to calculate the DQE from the statistics of individual events [6]. This is illustrated in Fig. 8 where the analysis of images obtained with an average dose of 1 electron per 100 pixels is presented.

As the MAPS detector is also capable of distinguishing individual electron events, it is possible to calculate the DQE in a similar way. This requires generating the probability distribution, ρ(E), for recording an incident electron with a signal E by measuring the response of single electron events in low dose images. From this probability distribution (which is known as the *pulse height spectrum*, *straggling function* or *Landau plot*) the DQE is obtained from the first $\left(M_{1}\right)$ and second $\left(M_{2}\right)$ moments using

(16) $$\mathrm{DQE}(0)=M_{1}^{2}/M_{2}$$

in which $M_{2}=\int E^{2}\rho (E)\;dE$ and $M_{1}=\int E\rho (E)\;dE$ is the mean response. Obtaining reliable results, especially for $M_{2}$, using this method requires accurate determination of ρ(E). This is complicated by the weak halo of signal in the surrounding pixels and the presence of multiple contributions per incident electron due to backscattering from the substrate. In particular an incident 300 keV electron can result in two distinct signals separated by over 200μm which must be identified and summed correctly.

## 5 More detailed results for film

**Fig. 9:**
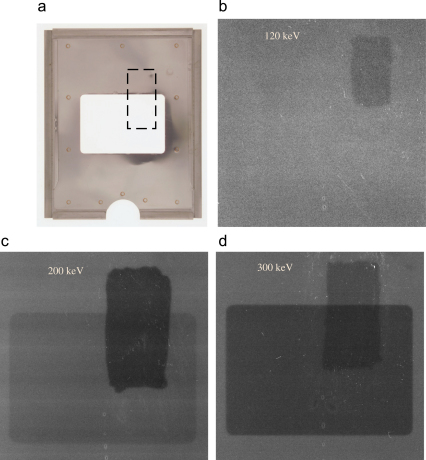
Images showing the effects at different incident electron energies of both electron backscattering from the metal film holder and from light generated within the plastic film backing due to the passage of high energy electrons in Kodak SO-163 film. (a) Photograph of a film holder showing the cut-away region in the centre. The area where black ink was applied to the back of the sheet of film to suppress light reflection is indicated by the dashed box. (b) Image taken with 120 keV electrons showing the area around the cut-away film holder. The OD in the area where the ink was applied is ∼2% lower (the developed film is lighter in the area where the ink was applied). At 120 keV there is no sign of any effects from the film holder. (c) Image taken with 200 keV electrons showing a reduction in OD of ∼9% with the ink and a ∼4% reduction where there is no film holder and so no electron backscatter from the film holder. (d) Image taken with 300 keV electrons showing a ∼10% reduction in OD with the ink and a ∼15% reduction in OD from the removal of electron backscatter.

The effects at higher voltages of electron backscattering from the metal in film holders is well known and because of this the results shown above for Kodak SO-163 film at 300 keV were obtained with a film holder in which the metal backing had been cut away from behind the area of interest. A less well known effect comes from light generated by incident electrons, presumably in the plastic support of the film. These two effects are illustrated in Fig. 9 which shows digitised images at 120, 200 and 300 keV obtained using film holders with a rectangular window cut out as illustrated in Fig. 9a. To show the effects of light a rectangular area on the back of the film, as indicated in Fig. 9a, was coated with black ink from a dry-marker pen before exposure and removed prior to development. Putting ink on the front of the film, or on the film holder, has no noticeable effect which indicates that the light is being reflected from the bottom surface of the plastic. At 120 keV, there is no sign of electron backscatter from the film holder but the area with the marker pen has a slightly (∼2%) lower OD. At 200 keV the light effect is more marked (∼9%) and it is also possible to see a small contribution (∼4%) from electrons backscattered from the holder. At 300 keV the light effect increases slightly (∼10%) but the effect from electron backscatter is now much greater (∼15%).

**Fig. 10:**
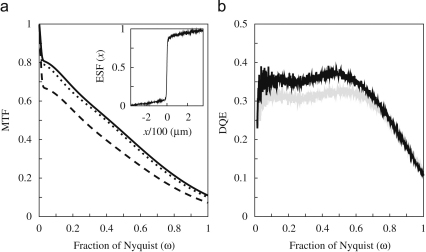
(a) The measured MTF at 300 keV for SO-163 film showing the effects of electron backscattering from the metal holder and light generated in the plastic backing: dashed line is the MTF obtained with an unmodified holder; the dotted line is with the metal backing removed; the solid line is with both the metal backing removed and ink applied to suppress light reflection off the bottom of the film. The inset in (a) shows the measured ESF used in calculating the solid line. (b) The measured DQE for SO-163 film as a function of spatial frequency with (grey) and without (black) the metal holder.

Both the reflected light and the electron backscatter contribute to a long range tail in the PSF of film and consequent rapid drop in the MTF at low spatial frequencies. The stochastic nature of the scattering of light and electron backscatter means that, unlike deterministic blur, the reduction in the MTF is not reflected in the NPS, and both effects will reduce DQE(ω) for ω≠0. This is illustrated in Fig. 10a which shows the measured MTF results at 300 keV obtained with a normal film holder, over a cut-away section, and over a cut-away section with black ink. The corresponding DQE(ω) results for a normal film holder and over a cut-away section are shown in Fig. 10b. The films used to calculate the DQE and MTF were taken with an OD of approximately 1 and digitised using 6μm steps on the MRC-LMB KZA scanner [32].

Even with the metal backing removed and ink suppressing the reflection of light, the ESF still shows a long range tail (see Fig. 10 a inset). Moreover this residual component is actually greater than that from both the backscattering of light and electrons from the film holder. Simulations presented in Section 6.1 confirm that the majority of this remaining tail is due to backscattering within the plastic base of the film.

**Fig. 11:**
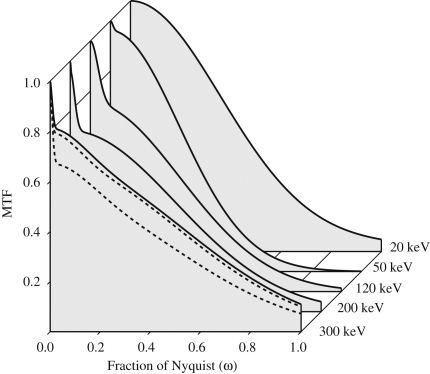
Comparison of measured spatial frequency variation of the MTF of SO-163 at 20, 50, 120, 200 and 300 keV. The images were scanned with a 6μm step. At 300 keV, results for a normal holder, cut-away holder and cut-away holder with light absorbing ink are shown. The MTF of the densitometer has not been corrected for and so the intrinsic MTF values for film at Nyquist will be about twice that shown here. Note how the minimum MTF at Nyquist occurs at the energy giving the maximum response per incident electron (see Fig. 7).

Fig. 11 shows the measured MTF of SO-163 film as a function of incident voltage. The images were scanned using the MRC-KZA scanner with a 6μm pixel. At low energies the MTF can be described with a single Gaussian. The width of this Gaussian depends on the spread of the incident electrons in the film emulsion as well as the densitometer optics and pixel size. The change in width with increasing energy of the incident electrons reflects the greater scatter associated with electrons penetrating further into the film's emulsion. Above the peak response at ∼50keV (see Fig. 7) the shape of the MTF changes to have distinct low and high spatial frequency components. The low spatial frequency component in the MTF comes from backscattered electrons. Initially the backscattering comes only from the plastic support but at higher energies (≥200keV) it can be boosted by the contribution from electron backscattering from the metal film holder. With increasing energy the range over which electrons can backscatter increases and this is reflected in a decrease in range of ω over which the backscatter term contributes to the MTF. The high spatial frequency component comes from the initial passage of the incident electron through the emulsion. The rate in fall-off with increasing spatial frequency of this term decreases with increasing energy as electrons experience fewer collisions, and hence less lateral scattering, whilst traversing the emulsion. At 300 keV, results are shown for a normal holder, a cut-away holder and cut-away holder with ink (see Fig. 10a). Note that with a cut-away holder, the backscatter contribution to the MTF at 300 keV is lower than at 200 keV. This is due to a greater fraction of the incident electrons being transmitted through the plastic support before having a chance to backscatter. The amount of backscatter could be further reduced by using a thinner plastic support.

## 6 Simulations

Monte Carlo simulations of electrons normally incident on film and the MAPS detector were carried out. The details of the Monte Carlo calculation are similar to those used previously to describe the Medipix2 detector [6] and follow the prescription given in the book by Joy [33]. The Mersenne-Twister pseudo-random number generator [34] was used in all the calculations.

In the simplest approach, the trajectories of electrons are calculated using a screened Rutherford cross-section to describe the elastic collisions and the continuous slowing down approximation (CSDA) to describe the energy loss due to inelastic collisions. In this approximation, directional changes are due solely to elastic collisions and energy is lost continually between elastic collisions at a rate determined by the Bethe approximation. Because of the light mass of electrons, calculations require relativistic effects to be taken into account even at 100 keV. While the CSDA should be adequate to describe average properties such as the MTF, it is not expected to give an adequate description of the DQE, which depends on the stochastic nature of the energy deposited in the detector. To investigate this, calculations for the MAPS detector in which both elastic and inelastic collisions are treated stochastically were carried out. In this approach, which we call full Monte Carlo (FMC), the inelastic mean free path and energy loss distribution are determined from inelastic cross-sections based on those supplied by Hans Bichsel [35]. The cross-sections include a realistic description of the various energy loss mechanisms in silicon. In order to simplify calculations the recorded signal is assumed to be proportional to the energy loss of an incident electron and simulations terminated when the incident electrons energy falls below 0.25 keV.

### 6.1 Simulation of film

The Monte Carlo model used in the simulations of SO-163 film consists of a 0.5μm layer of gelatin, 8μm of emulsion, 178μm of polyester and a 200μm layer of stainless steel. As we do not know the precise make up of the emulsion, we have assumed it to be 60% gelatin and 40% silver bromide by volume, and so have a density, average atomic number and average atomic weight of 3.4, 11.2, and $24.4\;g/{\mathrm{cm}}^{3}$, respectively. The emulsion layer is partitioned into cubic voxels on a 0.2μm grid and filled with non-overlapping grains consisting of neighbouring voxels. During the simulation of the trajectory of an incident electron the emulsion is assumed to be uniform and the energy deposited within any voxel recorded. For each incident electron, the sum of the energy deposited within the volume occupied by a grain is calculated and the grain made developable, and subsequently treated as opaque, if the energy exceeds a given threshold. The final image is calculated using the Nutting formula [9] in which the optical density is proportional to the sum of cross-sectional area of developed grains using

(17) $$\mathrm{OD}=\log_{10}e\frac{n_{A}\overset{¯}{a}}{A}=0.434\frac{n_{A}\overset{¯}{a}}{A}$$

in which $n_{A}$ is the number grains of effective mean area $\overset{¯}{a}$ in an area A. The choice of grain size and threshold energy for development of the grains was guided by comparison of calculated and observed variation in OD as a function of both electron dose and incident energy (see Fig. 7). In the calculations presented, a threshold energy of 0.5 keV is used and grains are taken to be identical and made up of all voxels within a radius of 2 voxels about a central voxel so that an individual grain consists of 33 voxels. Use of the CSDA approximation and fixed alignment of the grains with respect to the incident electrons leads to a strong peak in the distribution of energy deposited in a grain corresponding to the initial rate of energy loss multiplied by the thickness of the grain. To avoid artefacts from this peak a random energy from a Gaussian distribution with width of 50 eV was added to the calculated energy. In order to generate an image that is incommensurate with the voxel grid, the contribution from each grain was also randomly shifted within the plane of the emulsion by up to one half of a voxel.

The maximum OD for current SO-163 film under the development conditions described in Section 3 was extrapolated from a plot of measured OD as a function of electrons per $\mu m^{2}$ and found to be 6.44 (slightly lower than the value of 7.86 given in Hahn [15]). Since the grains are taken to occupy 40% of the 8μm thick emulsion there are 12.1 grains per $\mu m^{2}$. To satisfy Eq. (17) each grain is therefore required to have an effective area of $1.22\;\mu m^{2}$, i.e., 2.35 times the cross-sectional area of an undeveloped grain. This factor reflects both the inadequacy of the simple Nutting formula (Eq. (17)) to describe absorption and multiple scattering of light from silver grains as well as differences between developed and undeveloped grains [9].

**Fig. 12:**
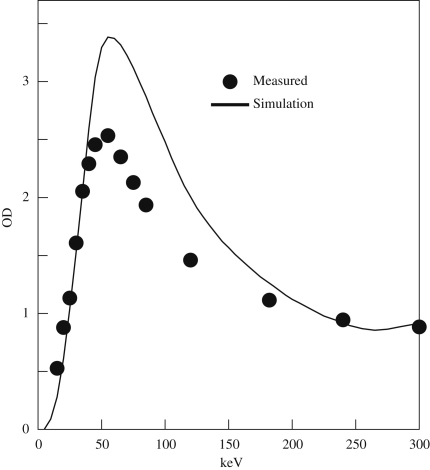
Comparison of measured and simulated OD in response to one electron per $\mu m^{2}$ as a function of incident energy for SO-163 film.

The measured variation in OD with incident energy is compared in Fig. 12 with the calculated variation obtained using the approximations described above. The initial slope and peak position do not depend strongly on choices of grain size and threshold energy.

**Fig. 13:**
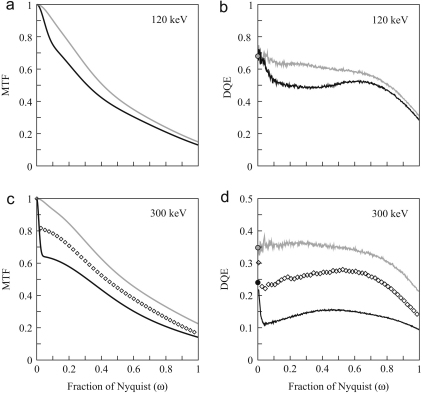
Monte Carlo simulation results for SO-163 film: (a) MTF at 120 keV; (b) DQE(ω) at 120 keV; (c) MTF at 300 keV; and (d) DQE(ω) at 300 keV. Results corresponding to SO-163 film in a normal film holder are shown in black. The effect of electron backscattering is illustrated by showing results obtained with a backless film holder using ♢ and for backthinned film (in which there is also no plastic support for the emulsion) in grey.

Using the model described above, simulations of flat field and knife edge images were carried out at both 120 and 300 keV. The resulting images were convolved with the response function of the densitometer and then analysed in the same way as experimental images using the methods described in Section 3 to obtain the MTF and DQE. Results of these calculations are presented in Fig. 13. Also shown are simulations of backthinned film, i.e., having no backing plastic or holder. As expected these do not show the rapid drop in MTF and DQE at low spatial frequency associated with the backscattering of incident electrons.

### 6.2 Simulation of the MAPS detector

**Fig. 14:**
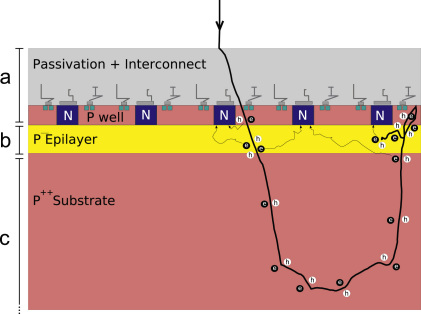
Schematic of MAPS CMOS detector. The pixel spacing is determined by the spacing between diodes formed by the N well doped areas indicated in blue. The division into three layers used in the simulations is indicated and consists of: (a) passivation and heavily doped wells; (b) sensitive layer consisting of the lightly doped epilayer; and (c) heavily doped substrate. The track of an incident electrons is shown illustrating the problem with backscattering from the substrate in a non-backthinned detector. The diffusive collection by the reverse biased N well diodes of mobile electrons generated in electron-hole pair excitations is indicated.

A schematic cross-section of a MAPS detector is shown in Fig. 14. The complication found with incident electrons of energies typically used in electron microscopy, from backscattering from the substrate, is also illustrated. The MAPS detector consists of a heavily p-doped substrate supporting a lightly p-doped epilayer onto which heavily doped n- and p-well areas are deposited. The n-well areas form reverse biased diodes whose voltages are set and readout with the aid of nMOS transistors fabricated on adjacent p-well areas. Further layers containing inter-layer dielectrics and the metallic interconnects are deposited on top of this and the whole detector is capped with a final passivation layer.

A MAPS detector works by measuring the change in voltage that occurs on discharge of the capacitor (associated with the reverse biased diode) by the collection of free charge carriers from the epilayer. In the present case, the charge carriers are electrons generated from electron-hole pair excitations created either thermally or by the passage of an incident charged particle. The thermal contribution limits the integration time between successive readouts, but as with CCDs it can be greatly reduced by working at lower temperatures. Charge carriers found in the epilayer can be generated both in the actual epilayer and in the substrate. The volume from which the latter contribution can come is limited by the higher doping, and subsequent short lifetime of minority carriers [36,37]. Once in the epilayer, electrons are trapped in the potential well resulting from the doping differential at the boundary. Electrons in the depletion region around the n-well diode will be collected with high efficiency. However this region only extends for about a micrometre and the majority of electrons collected must first diffuse within the epilayer in order to be collected by a diode. The minority carrier lifetime in the epilayer allows efficient collection but at the expense of pixel crosstalk.

In the detection of light each incident photon only results in the order of one electron-hole pair and so pixel crosstalk acts as stochastic scattering which will dramatically reduce DQE(ω) [12,38]. With high energy electrons, each incident electron results in the production of several hundred epilayer electrons. As a result pixel crosstalk will resemble deterministic diffusion and is not therefore be expected to dramatically reduce DQE(ω) [38]. The large number of electron-hole pairs produced per incident electron does however limit the dynamic range of a MAPS detector.

The Monte Carlo method is used here to simulate the trajectory and energy loss of electrons normally incident on the detector. A realistic simulation of a MAPS detector has to contend both with the charge generation and collection in a complex semiconductor structure as well as random initial conditions generated by the energy loss along the stochastic trajectory of the incident high energy electron. In the present work, the structure of the detector is simplified to three layers, all taken to be silicon, representing the passivation, sensitive epilayer and substrate. Calculations for a totally backthinned detector, in which the substrate has been removed, will also be presented.

For simplicity the signal is assumed to be proportional to the energy lost within the sensitive layer by the incident electron. The actual thickness of the sensitive layer is not well defined due to doping profiles in manufacture and the contributions from charge generated outside the actual epilayer. The signal is also not simply given by the energy loss and in order to explain the observed DQE we find that this assumption must be modified (see below). The effect of electron diffusion parallel to the epilayer is taken into account by convolving the deposited energy distribution with a Gaussian. The width of this distribution is estimated empirically.

**Fig. 15:**
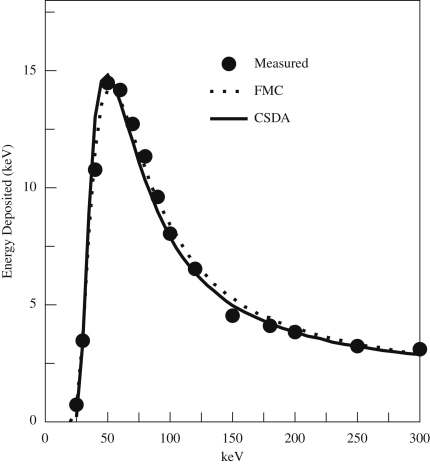
Calculated average energy per incident electron deposited within the sensitive layer of the MAPS CMOS detector as a function of incident energy using—CSDA approximation and … the FMC. The measured results, indicated by the solid circles, have been scaled from the raw ADC values.

The thickness of the capping and sensitive layers of the MAPS used in this work are both believed to be ∼5μm [7]. The variation in signal with incident energy enables us to refine these values. The minimum detectable energy determines thickness of the capping layer while the peak position and downward slope, fix the thickness of the sensitive layer. As illustrated in Fig. 15, values of 5 and 4μm for the capping and sensitive layers, respectively, give good agreement with the observed behaviour in both CSDA and FMC calculations. While the onset and peak position have been refined by parameter adjustment, the ratio of the peak height to that at 300 keV was not. The fact that it comes out close to the measured value gives confidence that the simulations are realistic.

**Fig. 16:**
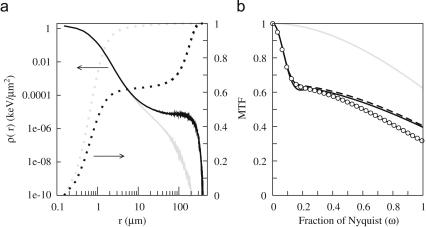
(a) Solid lines show the calculated deposited energy density as a function of radius for incident 300 keV electrons on the 3-layer (black) and 2-layer, i.e., backthinned, models for the MAPS detector. The dotted lines show the cumulative integrals of the corresponding point spread functions as a function of radius. By definition these must equal unity for large radius. (b) Comparison of measured (∘) and various calculated MTF results for MAPS detector at 300 keV. The MTF calculated from the PSF and an edge simulation are shown as solid and dashed lines, respectively. The backthinned MTF is shown in grey. The calculated edge MTF multiplied by $\exp(-0.26\omega^{2})$ is indistinguishable from the measured MTF.

Two methods were used to calculate the MTF. One mirrored the approach used experimentally and simulated the response of a detector to a uniform beam obscured by a knife edge at a small angle to the pixel grid. The resulting image was analysed in the same way as the experimental data. The other method involved first calculating the point spread function, PSF, and then taking the Fourier transform of this. To compare this result with the previous one, the resulting MTF must be multiplied by the sinc(πω/2) pixel modulation factor. To calculate the point spread function, electrons were incident on a single point and the circular average about this point of the energy deposited in the sensitive layer calculated. The results of PSF calculations at 300 keV are illustrated in Fig. 16a where the circular average of the radial energy density is shown from the CSDA calculation both with and without substrate. For clarity the results are plotted on a log–log plot. Up to 5μm the energy distributions in detectors with and without substrate are almost identical. Beyond 5μm the two curves diverge with the non-backthinned detectors energy density gaining an extra contribution from backscattered electrons so that the total integrated energy is 1.87 keV for the backthinned and 2.86 keV for the non-backthinned detectors. On a log–log plot the results of the FMC calculations look almost identical with the corresponding integrals being 1.86 and 2.95 keV, respectively. The FMC results do however extend to slightly greater distances and this subtle difference leads to a slightly greater initial drop in the MTF. Also plotted in Fig. 16a are the cumulative integrals of the PSF as a function of radius. By definition this is 1.0 at large distances. For the backthinned detector, it is notable that the PSF is almost entirely contained within a disk of radius 5μm.

The measured and various calculated MTF results are shown in Fig. 16b. The calculated MTF obtained using a simulated knife edge or via the PSF agree. The small dip in MTF(ω) calculated from the PSF at around ω=0.17 is an artefact from the Fourier transform of sharply peaked PSF. Multiplying the calculated MTF by $\exp(-0.26\;\omega^{2})$ maps it onto the experimentally measured MTF. The multiplication factor corresponds to describing the epilayer diffusion by a Gaussian with a 1/e width of 0.32 pixels, i.e., 8.1μm for a 25μm pixel. With a large pixel, the effect of this diffusion on the MTF at Nyquist MTF is relatively small. However, in a detector with smaller pixels the effect would be far greater. The shape of the MTF curve mirrors that of the cumulative integral over the PSF indicated as dotted lines in Fig. 16a with the contribution from the long range part in Fig. 16a equalling that from the corresponding initial drop in the MTF shown in Fig. 16b. The calculated MTF of the backthinned detector is essentially the sinc function of a perfect pixel detector. A real detector would also have to include the epilayer diffusion term. Using the 8.1μm diffusion width found above reduces the MTF at Nyquist from 63% to 49%. The shape of the MTF curve from the FMC calculation is the same as that from the CSDA calculation but the larger long range contribution leads an initial drop in the MTF to more like 60% rather than 65% shown for the CSDA calculation.

**Fig. 17:**
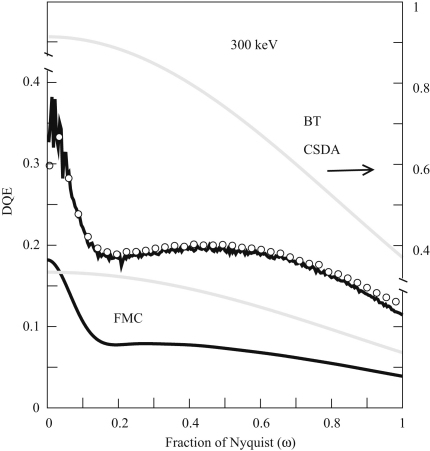
Comparison of measured and calculated spatial frequency variation of the DQE for a MAPS detector at 300 keV. The measured DQE is indicated by the circles (∘) while the results of calculations using both the CSDA and FMC approximations for non-backthinned (black) detectors and backthinned (grey) detectors are shown. The non-backthinned CSDA calculation results are in very good agreement with the measured results. The backthinned CSDA are within ∼90% of those for a perfect and plotted against the right hand axes. The corresponding FMC results are less than the measured results. Note that the value of DQE(0) for a backthinned detector is less than that for a non-backthinned detector. The backthinned detectors do not show the drop with increasing ω due to backscattering.

Simulation results, based on both the CSDA and FMC approximations, for DQE(ω) of a MAPS detector operating with 300 keV electrons are compared with the measured results in Fig. 17. As for the MTF calculations, two methods were used to calculate DQE(ω). One method mimicked the approach used experimentally and calculated the power spectra from simulation of uniformly illuminated areas. The other approach followed the method used by Meyer and Kirkland [39], and summed the power spectra of individual events. Both methods give similar results but in using the power spectra of individual events it is important to take into account aliasing and the sinc(πω/2) pixel integration weighting factor.

**Fig. 18:**
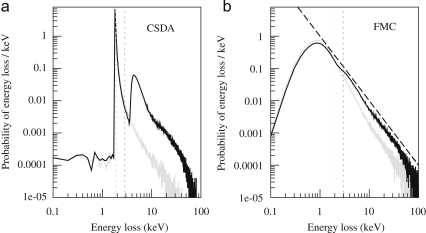
Calculated probability distributions obtained using the (a) CSDA and (b) FMC models for depositing energy E in a MAPS detector by an incident 300 keV electron. The results for backthinned (grey) and non-backthinned (black) detectors are shown. The dotted vertical lines indicate the position of the corresponding mean energy loss. The dashed line in the FMC calculation indicates the $1/E^{2}$ energy dependence of the inelastic Rutherford cross-section.

Naively the CSDA calculation would be expected to underestimate the variability in energy deposition and so overestimate the DQE. In fact the CSDA results agree remarkably well with the measured results while the corresponding FMC results are lower than the measured results. To understand this, the probability distribution, ρ(E), for an incident electron to lose energy E was calculated. As noted in Section 4, this distribution is variously known as the pulse height distribution, straggling function or Landau plot and with Eq. (16) can be used to calculate DQE(0). Results for ρ(E) for both non-backthinned and backthinned detectors obtained using the CSDA approximation are presented in Fig. 18a. For a backthinned detector ρ(E) is very strongly peaked at the mean energy loss (corresponding approximately to the initial rate of energy loss multiplied by the thickness of the sensitive layer). As a result $M_{2}\approx M_{1}^{2}$ and the value of DQE(0) calculated using Eq. (16) can approach unity. For the calculation presented in Fig. 18a, $M_{1}=1.87$ and $M_{2}=4.4$ so that DQE(0)=0.8. Note that the low energy tails of ρ(E) in a CSDA calculation comes from electrons that only travel a short distance into the sensitive layer before being backscattered out of the detector while the high energy tail comes from electrons that are scattered by longer distances within the sensitive layer. The calculated ρ(E) obtained using the CSDA for a non-backthinned detector has two distinct peaks. The lower energy peak comes from electrons which only travel through the epilayer once, while the higher energy peak comes from electrons that are backscattered from the substrate. The fact that the higher energy peak is at more than twice the energy of the first peak reflects the greater rate of energy loss from the less energetic backscattered electrons. The presence of this backscatter leads to both an increase in the average energy and in the variance of the energy deposited per incident electron in the sensitive layer. The increase in variance will in general lead to a decrease in DQE(ω) but the additional contribution to the average signal helps suppress the decrease as ω→0. As in the case of film, the presence of backscattering leads to a drop in DQE(ω) with increasing ω. As the ratio of pixel size to range of the backscattering from the substrate is larger than in film, the drop in DQE(ω) is spread over a wider range in ω. The strongly peaked shape of ρ(E) obtained using the CSDA approximation is not actually a good description of what is observed [8,26] and therefore the excellent agreement with experiment shown in Fig. 17 must be treated as fortuitous. The shape of ρ(E) obtained with the FMC simulations of a non-backthinned detector, as shown in Fig. 18b, gives a much better description of that observed experimentally. As the mean ionisation potential used in the CSDA, and corresponding value obtained from the inelastic cross-section used in the FMC calculation agree, i.e., 174 eV [35], it is not surprising that both calculations result in similar expected average energy loss. In the FMC calculation the average expected energy loss of ∼3keV occurs well above the most likely energy loss of ∼1keV.

Unlike with the CSDA, the calculated shape of ρ(E) for a backthinned detector using the FMC approximation is very similar to that of the non-backthinned detector. This reflects the intrinsic variability using FMC of both the amount of energy that can be lost in an individual inelastic event and the number of inelastic events per unit length. As a consequence FMC calculations do not show the dramatic increase in DQE(0) with backthinning seen in the CSDA calculations. In fact the lower mean and intrinsic variability results in the calculated DQE(0) actually being slightly lower for the backthinned detector (Fig. 17). However, backthinning removes the drop in DQE(ω) with increasing ω associated with backscatter of incident electrons from the substrate.

**Fig. 19:**
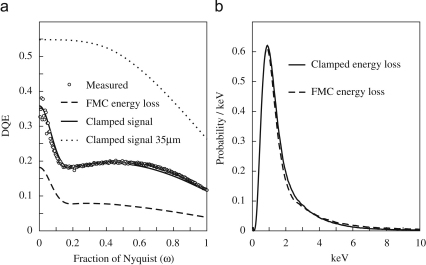
(a) Comparison of the measured (∘)DQE(ω) at 300 keV of the MAPS detector with the results of calculations using the FMC (dashed) and the clamped FMC approximations (solid) described in text. The dotted line gives the calculated FMC clamped DQE(ω) for a detector backthinned (by the removal of the substrate) to a total thickness of 35μm. (b) Comparison of the probability distributions for the signal collected from a single incident 300 keV electron, obtained using the FMC approximation energy loss and the corresponding clamped FMC approximation.

As indicated in Fig. 18b, the calculated probability in a FMC calculation for an incident 300 keV electron to lose energy E in the sensitive layer of a MAPS detector follows approximately the $1/E^{2}$ behaviour of the Rutherford inelastic cross-section. The high energy tail for ρ(E) is therefore very important in determining DQE(0). By limiting the signal recorded in the detector, rather than simply assuming it is proportional to the energy loss, we can suppress the contribution from the high energy tail of ρ(E) and increase the predicted DQE(0). Limiting the total signal from an incident electron would be inconsistent with the observed linearity in response as a function of incident electrons. However it is possible to obtain a higher DQE(0) by limiting the response locally in the sensitive volume. This is illustrated in Fig. 19 which shows the DQE(ω) and comparison of energy loss and detected signal distributions for what we will call a clamped FMC calculation. In this the sensitive layer is divided into cubic voxels and the energy loss from an incident electron turned into a spatial density distribution by convolving each inelastic event of the FMC calculation with a Gaussian point spread function. The contribution to the signal from each voxel is taken to be the integral of the energy density distribution within the voxel but clamped to a fixed maximum value. This model has three parameters: the width of the Gaussian point spread; the size of the cubic voxels; and the maximum energy contribution. Using a broad Gaussian and a small energy maximum it is possible to mimic the CSDA result, though with a lower average signal per incident electron. The calculations presented in Fig. 19 used a Gaussian with 0.5μm width, 0.782μm voxel dimension and maximum energy per voxel of 0.30 keV. The limiting energy corresponds to an energy density of $0.63\;\mathrm{keV}/\mu m^{3}$ and, assuming it takes 3.6eV per electron-hole pair, represents a limiting electron-hole pair density of $1.7\times 10^{14}\;{\mathrm{cm}}^{3}$. As shown in Fig. 19a the effect of clamping the local response is to increase the calculated DQE(ω). Note that the Gaussian blur introduced in this calculation also reduces the noise aliasing fall-off of DQE(ω) relative to that of the FMC calculation. With the parameters given above, the calculated value of DQE(0) increases as more of the substrate is removed. This is illustrated in Fig. 19a by the calculated DQE(ω) for a detector that has been backthinned to have a total thickness of 35μm. For a totally backthinned detector the corresponding value of DQE(0) is 0.7.

Fig. 19b, compares the calculated probability distributions for energy loss and detected signal in the sensitive layer for an incident 300keV electron. Superficially the curves look very similar. However, in the clamped FMC curve some of the high energy weight has been moved to lower energies. As a result the average signal per incident electron is 72% of that for the original FMC calculation.

The clamped FMC calculation is able to fit the observed DQE(ω) but introduces a number of adjustable parameters. Physically the approximation might be justified in the absence of an applied drift field by faster electron-hole recombination in areas with higher carrier density. It would, however be interesting to see how this approximation compares with more sophisticated calculations.

## 7 Extrapolations and discussion

The results presented here confirm in theory and practice that film is a remarkably good detector for use in electron microscopy. Its performance at 300 keV can be enhanced by using cut-away backless film holders, a thinner plastic matrix, a slightly thicker emulsion and an anti-reflective coating on the back of the plastic support. In this respect the reformulation by Kodak about 5 years ago of SO-163 film to have a thinner emulsion produced a higher MTF but at the cost of a slight drop in DQE at 300 keV. Even if film were made perfect there would still be a desire for the immediate feedback of electronic detectors. We can therefore ask what must be done to improve the performance of the electronic detectors. The goal should be to construct a large area electronic detector with a DQE better than film at 300 keV up to the Nyquist resolution limit, along with much lower noise level and immediate readout.

The DQE(0) of the TVIPS CCD is better than that of film but with increasing spatial frequency DQE(ω) drops below that of film and this is most notable at high electron energy. Part of this drop is due to the contribution from backscattered electrons but another important contribution to the decrease at Nyquist also comes from the finite numerical aperture of the optical coupling and light scattering within the phosphor. Because of the small number of photons per incident electron this scattering is stochastic, which increases the noise level and degrades the higher spatial frequency DQE. This should be contrasted with what happens with the limited numerical aperture of a densitometer lens where the optical resolution does not dramatically decrease the DQE, due to the enormous number of photons involved. The electron backscatter term can be minimised by supporting the phosphor on a thin mylar sheet and using a lens-coupled CCD. However, in order to do this the efficient optical coupling that can be achieved with the fibre optic has to be sacrificed. To achieve similar optical coupling with a free standing phosphor places severe demands on the quality of the lens needed such as with the Gatan UltraCam [11].

Hybrid pixel detectors, such as the Medipix2, have difficulty at higher energies, e.g., 300 keV, because of the thickness of the sensor and resulting inability to localise the signal from an incident electron. At 120 keV the Medipix2 is a very good detector and its intrinsic parallel counting together with promised improvements could make it even better in the future. Hybrid pixel detectors operating in event counting mode, do however have a unique advantage in having no noise. This allows an image to be taken as either a single frame or built up in a series of frames.

A MAPS detector shares the features that make film such a good detector for low dose electron microscopy, in particular having a thin sensitive layer through which the passage of individual electrons can be detected and in which the incident electrons leave only a small fraction of their initial energy. A backthinned, radiation hardened, MAPS detector has the potential to deliver an electronic detector for use in electron microscopy whose DQE is as good as, or better than, that of film over all spatial frequencies. However, the DQE is limited by the intrinsic variability in the amount of energy deposited in a thin layer and the MTF limited by both epilayer diffusion and intrinsic sinc(πω/2) response of a pixel detector. It is possible to overcome both the limitations on the DQE and MTF by working in an electron counter mode. MAPS detectors are capable of detecting individual incident electrons. Identifying and counting events would thus create a noiseless detector without the reduction in DQE associated with variable energy deposition. The simulations presented in Section 6.2 also indicate that the intrinsic lateral spread of deposited energy associated with an incident 300 keV electron in a backthinned detector is only a few micrometres. The deterministic blur associated with epilayer diffusion will distribute this resulting signal over several pixels and from the relative signal in adjacent pixels the arrival position of an incident electron can be inferred to sub-pixel accuracy. The resulting smaller effective pixel dimension reduces the sinc(πω/2) damping associated with the original Nyquist frequency and leads to both improved MTF(ω) and DQE(ω). In order to use a MAPS detector in counter mode it must be capable of working at high frame rates as the total number of events per frame is limited by the necessity to distinguish individual events in a given frame and the total acquisition time for an image is limited by complications associated with sample drift. On the assumption that it is possible to identify individual events at a dose of one electron per 400 pixels, a usable detector that could acquire a low dose cryoEM image in 2 s with approximately 20 electrons per pixel, would have to operate at ∼4kHz. Such high frame rates give an opportunistic improvement in radiation hardness but the settling time (of a few μs) in current MAPS designs would not allow a detector operating at these frame rates to have more than a few hundred rows. As columns can be read out independently there is no intrinsic limit on their number, beyond those to do with fabrication, space and connection to analogue to digital converters. Assuming a detector can be built with more rows by having faster settling time and possibly multiple readout channels per column, one still has to contend with digitising and processing potential hundreds of gigabytes per second. Unfortunately, a large area electron counter of this type is still some way in the future.

Finally, although the MAPS detector appears to be most promising, it must be both backthinned and radiation hardened to achieve its full potential. A backthinned MAPS detector with a pixel size of 25μm (possibly slightly smaller) and with the lowest noise, highest sensitivity readout possible would offer a combination of excellent DQE, good MTF and reasonably good signal-to-noise ratio at low doses. If this could be designed to tolerate several MRad and have 4k×4k pixels, it would be the detector of choice for most low dose electron microscopy—until electron counters, which have no noise, can be improved to offer large area sensors with even better MTF and DQE at high energy.
